# Prediagnosis of Obstructive Sleep Apnea via Multiclass MTS

**DOI:** 10.1155/2012/212498

**Published:** 2012-04-01

**Authors:** Chao-Ton Su, Kun-Huang Chen, Li-Fei Chen, Pa-Chun Wang, Yu-Hsiang Hsiao

**Affiliations:** ^1^Department of Industrial Engineering and Engineering Management, National Tsing Hua University, Hsinchu 30013, Taiwan; ^2^MBA Program in International Management, Department of Business Administration, Fu Jen Catholic University, New Taipei City 24205, Taiwan; ^3^Department of Otolaryngology, Cathay General Hospital, Taipei 10630, Taiwan; ^4^Quality Management Center, Cathay General Hospital, Taipei 10630, Taiwan; ^5^Fu Jen Catholic University, School of medicine, New Taipei City 24205, Taiwan; ^6^Information and Communications Research Laboratories, Industrial Technology Research Institute, Hsinchu 31040, Taiwan

## Abstract

Obstructive sleep apnea (OSA) has become an important public health concern. Polysomnography (PSG) is traditionally considered an established and effective diagnostic tool providing information on the severity of OSA and the degree of sleep fragmentation. However, the numerous steps in the PSG test to diagnose OSA are costly and time consuming. This study aimed to apply the multiclass Mahalanobis-Taguchi system (MMTS) based on anthropometric information and questionnaire data to predict OSA. Implementation results showed that MMTS had an accuracy of 84.38% on the OSA prediction and achieved better performance compared to other approaches such as logistic regression, neural networks, support vector machine, C4.5 decision tree, and rough set. Therefore, MMTS can assist doctors in prediagnosis of OSA before running the PSG test, thereby enabling the more effective use of medical resources.

## 1. Introduction

According to the National Institutes of Health, 50–70 million Americans are affected by chronic sleep disorders and intermittent sleep problems that can significantly diminish health, alertness, and safety. Untreated sleep disorders have been linked to hypertension, heart disease, stroke, depression, diabetes, and other chronic diseases. Recently, the Institute of Medicine in its report estimated that sleep disorders and sleep deprivation constitute an inadequately addressed public health problem, and “hundreds of billions of dollars a year are spent on direct medical costs related to sleep disorders, such as doctor visits, hospital services, prescriptions, and over-the-counter medications.” According to the National Highway Traffic Safety Administration, drowsy driving claims more than 1,500 lives and causes at least 100,000 motor vehicle crashes each year [1].

Polysomnography (PSG) is traditionally considered an established and effective diagnostic tool providing information on the severity of obstructive sleep apnea (OSA) and the degree of sleep fragmentation. However, the PSG method is time consuming and labor intensive [2, 3], requiring overnight evaluation in a sleep laboratory, dedicated systems, and attending personnel. Accurate identification of an apnea event requires the direct measurement of upper airway airflows and respiratory efforts. Therefore, the development of a simple and effective predictive method for OSA diagnosis is important.

There are many inspections for OSA methods, including the O_2_ Pulse Oximeter, the body mass index (BMI), and the two-stage method (BMI-attached O_2_ Pulse Oximeter and questionnaire-attached O_2_ Pulse Oximeter). However, patients are still required to wear the O_2_ Pulse Oximeter overnight, which is very inconvenient for them [4–6].

Mahalanobis-Taguchi System (MTS) is a collection of methods that was proposed as a forecasting and classification technique using multivariate data developed by Dr. Taguchi [7, 8]. MTS integrates Mahalanobis distance and Taguchi's robust engineering. Mahalanobis distance is used to construct a multidimensional measurement scale and to define a reference point of the scale with a set of observations from a reference group. Taguchi's robust engineering is applied to determine the important features and then optimize the system. Thus far, MTS has been successfully used in various applications [9–13].

Multiclass Mahalanobis-Taguchi system (MMTS) breaks the limitation of MTS, in which only one Mahalanobis space is constructed for one problem and establishes an individual Mahalanobis space for each class to accomplish multiclass classification and feature selection tasks simultaneously. MMTS also inherits the robustness of classification from MTS [13]. The classification capability and feature selection stability of MMTS were both confirmed [14].

Therefore, this study used MMTS for OSA prediction to provide a convenient and fast prediction method. A comparison was also made between MMTS and other methods, including logistic regression (LR), back propagation neural network (BPN), learning vector quantization (LVQ), support vector machine (SVM), C4.5 decision tree, and rough set (RS).

## 2. Materials and Methods

Following the approval from the Cathay General Hospital, Taipei, Taiwan, this study gathered 124 subjects (90 men and 34 women) who were referred for clinical suspicions of OSA from October 2007 to July 2008. The patients were consecutively recruited from the outpatient clinic and taken through data preprocessing to prepare for the training and the testing data sets. Inconsistent data were deleted, and missing values in the analysis were ignored, leaving 86 subjects (62 diseased and 24 nondiseased) for our analysis.

The collected OSA data had 12 attributes, including anthropomorphic measurements (i.e., age, gender, height, weight, body mass index (BMI)), systolic blood pressure (SBP), diastolic blood pressure (DBP), frequency of desaturation (DI3, DI4), frequency of paroxysmal leg movements per hour (PLM), and questionnaire measurements (ESS, SOS). The explanations for each attribute are presented in Table 1. 

MMTS, which was developed by Su and Hsiao as a diagnostic and forecasting technique, uses multivariate data developed according to the MTS framework. It is used for simultaneous multiclass classification and feature selection. MMTS comprises four main implementation stages: construction of a full-model measurement scale with Mahalanobis space of each class as the reference; validation of the full-model measurement scale; feature selection; future prediction with important features.

### 2.1. Stage 1: Construction of a Full-Model Measurement Scale with Mahalanobis Space of Each Class as Reference

In this stage, the problem and all related features are defined, representative examples are collected to construct the individual Mahalanobis space for each class, and a full-model measurement scale is established. To enhance accuracy in constructing the measurement scale, the Gram-Schmidt orthogonalization process is applied to eliminate multicollinearity from among the features, making the covariance matrix almost singular and the inverse matrix invalid.

Assume that there are *k* classes in a *d*-dimensional space. For each class *C*_*i*_(*i* = 1, 2,…, *k*), the examples sampled from its population are defined as “normal” while the examples coming from other *k* − 1 classes are defined as “abnormal.” The Mahalanobis space MS_*i*_ is formed by the *n*_*i*_ normal examples sampled from *C*_*i*_. *A*_1^(*i*)^_^(*p*)^, *A*_2^(*i*)^_^(*p*)^,…, *A*_*d*^(*i*)^_^(*p*)^ denote the standardized feature vectors of MS_*i*_ standardized by the feature means and standard deviations of MS_*p*_. The Gram-Schmidt feature vectors of MS_*i*_  orthogonalized on the basis of MS_*p*_, that is, *U*_*l*^(*i*)^_^(*p*)^, are sequentially constructed from *l* = 1 to *l* = *d* by the following Gram-Schmidt setting:

(1) $$U_{l^{\left(i\right)}}^{\left(p\right)}=A_{l^{\left(i\right)}}^{\left(p\right)}-\overset{l-1}{\underset{q=1}{\sum }}t_{lq^{\left(p\right)}}\times U_{q^{\left(i\right)}}^{\left(p\right)},$$

where *A*_*l*^(*i*)^_^(*p*)^ is the *l*th feature vector of  MS_*i*_  standardized by MS_*p*_, *U*_*q*^(*i*)^_^(*p*)^ is the Gram-Schmidt vector of the *q*th feature of MS_*i*_ orthogonalized on the basis of MS_*p*_, and *t*_*lq*^(*p*)^_ is the Gram-Schmidt coefficient of MS_*p*_ and is set as follows for *l* = 1, 2,…, *d*, *q* = 1, 2,…, *l* − 1:

(2) $$\begin{array}{c} t_{lq^{\left(p\right)}}=\frac{A_{l^{\left(p\right)}}^{{\left(p\right)}^{T}}U_{q^{\left(p\right)}}^{\left(p\right)}}{U_{q^{\left(p\right)}}^{{\left(p\right)}^{T}}U_{q^{\left(p\right)}}^{\left(p\right)}}, \end{array}$$

where  *A*_*l*^(*p*)^_^(*p*)^ is the *l*th standardized feature vector of MS_*p*_ and  *U*_*q*^(*p*)^_^(*p*)^ is the Gram-Schmidt vector of the *q*th feature of MS_*p*_.

The Mahalanobis distance from any example *r* to *C*_*i*_ can be calculated using the Gram-Schmidt orthogonalization process as follows. First, the features in example *r* are standardized using the feature means and standard deviations of MS_*i*_. Next, the Gram-Schmidt coefficients of MS_*i*_ are employed to perform the Gram-Schmidt orthogonalization process on the standardized features of example *r*. The Mahalanobis distance from example *r* to *C*_*i*_, that is, MD_*r*_^(*i*)^, using the Gram-Schmidt orthogonalization process is calculated as the following equation:

(3) $${\text{MD}}_{r}^{\left(i\right)}=\frac{1}{d}\times \overset{d}{\underset{q=1}{\sum }}\frac{u_{rq}^{{\left(i\right)}^{2}}}{\zeta_{q^{\left(t\right)}}^{2}},$$

where *d* is the number of features, *u*_*rq*_^(*i*)^ is the Gram-Schmidt vector of the *q*th feature in example *r* processed by M*S*_*i*_, and *ζ*_*q*^(*i*)^_ is the standard deviations of *U*_*q*^(*i*)^_^(*p*)^ for *p* = *i*.

For the *n*_*i*_ normal examples in M*S*_*i*_, their Mahalanobis distances are to *C*_*i*_ (*i* = 1, 2,…, *k*) using the Gram-Schmidt orthogonalization process. With these Mahalanobis distances, the center point and the unit distance for each class can be defined, by which the reference base for the measurement scale is determined.

### 2.2. Stage 2: Validation of the Full-Model Measurement Scale

In this stage, the effectiveness of discrimination among different classes is validated through the full-model measurement scale. Therefore, the Mahalanobis distance to each Mahalanobis space is calculated for each example. The measurement scale is then validated by examining the reparability of the Mahalanobis distances corresponding to the examples with different classes.

For *C*_*i*_, *i* = 1, 2,…, *k*, the corresponding abnormal examples from the other *k* − 1 classes are used to validate the measurement scale. To do so, the Mahalanobis distances from the abnormal examples to *C*_*i*_ should be computed using (3). According to the MTS theory, the Mahalanobis distances of abnormal examples will be much larger than those of normal examples if the measurement scale is good. However, for *C*_*i*_, *i* = 1, 2,…, *k*, if there is no significant difference between the normal and abnormal Mahalanobis distances, then the constructed Mahalanobis space cannot suitably represent the corresponding real normal condition. Moreover, we should return to the beginning of the whole problem and perform some checks on the completeness of considered features or on the representative of the collected examples used to construct Mahalanobis space.

### 2.3. Stage 3: Identification of the Important Features

In this stage, orthogonal arrays and signal-to-noise ratio are used to identify the important features for multiclass classification.

Each of the original *d* features is first set with two experiment levels. Level 1 includes the feature in constructing the Mahalanobis space while Level 2 excludes the feature. Afterward, an appropriate orthogonal array is chosen, and the *d* features are assigned into different columns of orthogonal array. Inside the orthogonal array, every row (run) presents a different level combination of features. For each run, the features with Level 1 are used to construct the Mahalanobis space for *C*_*i*_, *i* = 1, 2,…, *k*. In addition, the MD_*j*^(*i*)^_^(*p*)^ for *j* = 1, 2,…,*n*_*i*_, *i* = 1, 2,…, *k*, and *p* = 1, 2,…, *k* are calculated according to (3) and are regarded as the output of each run. Thus, in each run, there will be *n*_*i*_ normal Mahalanobis distances and ∑_*q*=1_^*k*^ *n*_*q*_ abnormal Mahalanobis distances produced for *C*_*i*_, where *q* ≠ *i*. When an example *r* comes from MS_*i*_, a high ratio MD_*r*^(*i*)^_^(*p*≠*i*)^/MD_*r*^(*i*)^_^(*p*=*i*)^ is expected. For this reason, the signal-to-noise ratio *η* corresponding to each run of orthogonal array is computed using the concept of the larger-the-better type and is defined using the following equation:

(4) $$\eta =\overset{k}{\underset{i,p=1}{\sum }}\left[-10\times \log_{10}\left(\frac{1}{n_{i}}\times \overset{n_{i}}{\underset{j=1}{\sum }}{\left(\frac{{\text{MD}}_{j^{\left(i\right)}}^{\left(p\neq i\right)}}{{\text{MD}}_{j^{\left(i\right)}}^{\left(p=i\right)}}\right)}^{-1}\right)\right]=\overset{k}{\underset{i,j=1}{\sum }}\eta_{ip},$$

where *n*_*i*_ is the number of examples in the Mahalanobis space MS_*i*_; MD_*j*^(*i*)^_^(*p*=*i*)^ is the Mahalanobis distance from the *j*th example in MS_*i*_ to class *C*_*p*_ and *p* = *i*; and MD_*j*^(*i*)^_^(*p*≠*i*)^ is the Mahalanobis distance from the *j*th example in MS_*i*_ to class *C*_*p*_ and *p* ≠ *i*.

For the *l*th feature, ${\overset{̅}{\text{SN}}}_{l}^{+}$ is used to represent the average signal-to-noise ratio of all runs including the feature, whereas ${\overset{̅}{\text{SN}}}_{l}^{-}$ represents the average signal-to-noise ratio of all runs excluding the feature. Independently evaluating the effect of each main factor is allowable because orthogonal arrays are used. Thus, the “effect gain” of each feature can be directly calculated using the following equation:

(5) $${\text{Gain}}_{l}={\overset{̅}{\text{SN}}}_{l}^{+}-{\overset{̅}{\text{SN}}}_{l}^{-}.$$

If the effect gain corresponding to a feature is positive, the feature may be important and may be considered as worth keeping. However, a feature with negative effect gain should be removed.

### 2.4. Stage 4: Future Prediction with Important Features

In this final stage, a reduced model measurement scale is constructed using the important features and then validated. A “weighted Mahalanobis distance” is employed to be the distance metric for classification. By simply classifying examples into the class with the minimum weighted Mahalanobis distance, the classification can be achieved.

The measurement scale is reconstructed using the feature subset *R* composed of *δ* important features identified in the third stage. This scale is called the “reduced model measurement scale.” Similarly, for  MS_*i*_, *i* = 1, 2,…, *k*, the validations of the scale should be applied using the corresponding abnormal examples to ensure that this reduced model has a good ability to discriminate among different classes. The weighted Mahalanobis distance weighing the different features in the Mahalanobis distance according to the corresponding effect gains obtained in the third stage is used for classification after the reduced model measurement scale is validated. The weighted Mahalanobis distance from any example *r* to *C*_*i*_ is computed through the following equation:

(6) $${\text{WMD}}_{r}^{\left(i\right)}=\frac{1}{\delta }\times \underset{l\in R}{\sum }w_{l}\times \frac{u_{rl}^{{\left(i\right)}^{2}}}{\zeta_{l(i)}^{2}},$$

where *δ* is the number of features in the reduced model, *w*_*l*_ is the weight of the *l*th feature in the reduced model, *u*_*rl*_^(*i*)^ is the Gram-Schmidt vector of the *l*th feature of example *r* processed by MS_*i*_ in the reduced model, and *ζ*_*l*(*i*)_ is the standard deviations of *U*_*l*^(*i*)^_^(*p*)^ in the reduced model for *p* = *i*.

The weight of the *l*th feature, that is, *w*_*l*_, in the reduced model can be acquired by normalizing the corresponding effect gain obtained in the third stage as

(7) $$w_{l}=\frac{{\text{Gain}}_{l}}{\sum_{l\in R}^{}{\text{Gain}}_{l}},$$

where Gain_*l*_ is the effect gain of the *l*th feature in the reduced model.

Based on this reduced model, a classification can be achieved by simply classifying examples into the class with minimum weighted Mahalanobis distance, and thus, the classification accuracy can be acquired. Importantly, a test experiment should be implemented using the unknown examples to confirm the classification ability of the reduced model.

**Figure 1:**
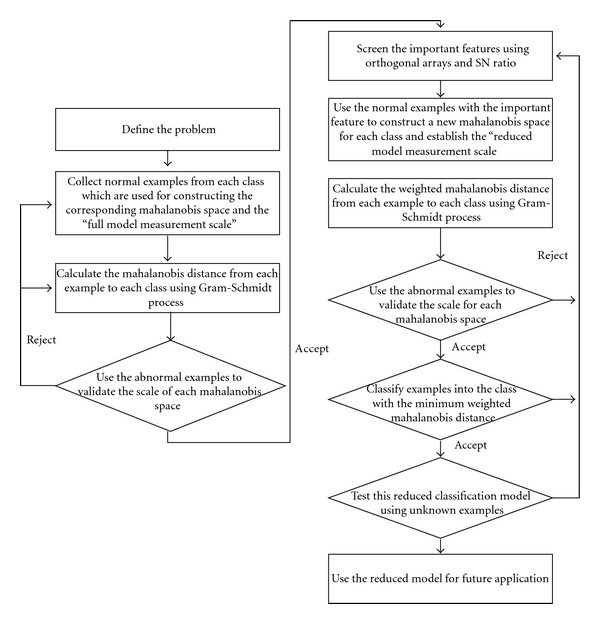
Procedure of implementing MMTS.

Note that the validation stage (Stage 2) plays an important role in MMTS algorithm. Stage 2 aims to check if the measurement scale is constructed well. That is, it is used to ensure that the measurement scale has the basic ability to discriminate the examples used to construct the Mahalanobis space and the examples out of the space. A valid measurement scale also implies that the important features of a problem have been considered and the representative examples have been collected for analysis. A comprehensive feature set and representative examples are prerequisites for establishing a good MMTS model. Moreover, Stage 3 of MMTS, the feature selection stage, is meaningless if valuable features are not considered and included at the beginning of problem analysis. Thus, the validation stage is also a way for checking the completeness of features and the representation of collected examples, and it is needed for ensuring the quality of the established MMTS model (Figure 1).

The four stages of implementing MMTS are shown in Figure 1. For details on MMTS, refer to Su and Hsiao [14].

## 3. Implementation

PSG, a multiparametric test used in sleep medicine, provides reliable data on OSA through comprehensive recordings of biophysiological changes that occur during sleep. It involves the following data: electroencephalogram (EEG), electrooculogram (EOG), electromyogram (EMG), heartbeat, and oximeter of the lobe. Scoring is accomplished through the Rechtschaffen method, which grades the severity of sleep apnea by the number of events per hour and is reported as a respiratory disturbance index (RDI). Patients were placed into four groups: the group with an RDI value <5 is normal; 5–15 is mild; 15–30 is moderate; >30 events per hour is characterized as having severe sleep apnea. In this study, MMTS was employed in the classification of OSA patterns.

To illustrate the effectiveness of MMTS for OSA prediction, comparisons were made between MMTS and other methods, including LR, BPN, LVQ, SVM, C4.5 decision tree, and RS. LR was first established as an analytical tool in epidemiology. It is used extensively in the medical and social sciences and has become the accepted “standard” in various research areas.

Artificial neural networks (ANNs) are computer programs modeled after the biological nervous system and are capable of recognizing complex patterns in data based on experience. These programs have been demonstrated as promising classification tools because their learning ability allows them to determine optimumn onlinear relationships between classes and to feature patterns from data sets. Both BPN and LVQ are common types of ANNs. On the other hand, SVMs have been successfully applied to classification and regression problems such as character recognition developed by Su and Hsiao [15]. A decision tree is a decision support tool that uses a tree-like graph or model of decisions and their possible consequences, including chance event outcomes, resource costs, and utility. The decision tree is the most efficient approach to addressing classification issues. The RS theory was introduced by Pawlak and is a mathematical tool. This theory provides a tool to mine knowledge as decision rules from a database or web-based information among others [16].

In this comparison, SVM was implemented using LIBSVM, which provides an efficient parameter selection tool using cross-validation through a parallel grid search performed under the kernel of the radial basis function type. Both BPN and LVQ are ANN models constructed for this study using the Professional II PLUS software. The parameters of BPN and LVQ contain the learning rate, momentum, and number of hidden nodes, which were optimized through trial and error to determine the combinations of the minimum root mean square errors. All the results of the C4.5 decision tree in this comparison were operated using the software tool see [17]. Finally, RSES and Weka software were used to implement RS and LR for classification problems, respectively. Statistical analysis was performed using SPSS v.14.0 (Statistical Package for Social Science, Chicago, IL).

## 4. Results

The subjects, including 66 men and 20 women, ranged in age from 11 to 78 years, with a mean age of 48.3 years (±11.87). Mean height was 165.97 (±7.34) mean weight was 69.05 (±11.31); mean BMI was 24.98 kg/m^2^ (±3.13); mean SBP was 124.64 (±17.62); mean DBP was 81.23 (±10.46), mean ESS score was 10.07 (±6.38) mean SOS score was 50.23 (±21.20), mean DI3 was 92.76 (±121.66), mean DI4 was 92.47 (±121.70); and mean PLM was 2.72 (±8.68). These results are summarized in Table 2.

This study separated the collected OSA data into two parts: Group I and Group II (Table 3). Group I was used to establish the model, whereas Group II was used to test the developed model. In the classification performance, the average classification rate of OSA obtained by each algorithm of Group II is shown in Table 4.

Table 4 shows the test results of the OSA data set. The obtained average accuracies of MMTS, LR, BPN, LVQ, SVM, C4.5 decision tree, and RS were 84.38%, 55.33%, 34.04%, 47.22%, 53.82%, 63.54%, and 13.20%, respectively. Results showed that MMTS had an accuracy of 84.38% on the OSA prediction, outperforming the other methods. Therefore, MMTS can be applied to assist doctors in foreseeing an OSA diagnosis before running the PSG test, thereby allowing a more effective use of medical resources.

## 5. Discussion

### 5.1. OSA

In this study, six important features, including age, weight, SBP, DBP, DI3, and DI4, are identified using MMTS. The other features not selected using MMTS include gender, height, BMI, ESS, SOS, and PLM. The following section briefly discusses these selected features.

Patients were placed into four groups: the group with an RDI value <5 is normal; 5–15 is mild; 15–30 is moderate; and >30 events per hour is characterized as having severe sleep apnea [18]. For the RDI value, higher is worse, lower is better.

In most studies, the age index is often used in the prediction model of OSA disease [19, 20]. OSA has two possible underlying causes: an anatomically vulnerable airway and neurologically unstable breathing control. As people grow older, their ability to control force in their airway weakens, thereby worsening their breathing. Thus, age is influential both neurologically and in the airway. This study found that hemodynamic parameters such as DBP, and SBP were more relevant to the development of OSA. For the Age, DBP and SBP, higher is worse, lower is better.

The ID3 and ID4 indices are the frequencies of desaturation (index <3% in an hour and index <4% in an hour, resp.). These indices can explain why there is more severe desaturation than the one predicted in alveolar hypoventilation, as demonstrated in OSA patients [4, 5]. In other words, oxygen desaturation occurs more often in proportion to the frequency of respiratory disturbances in OSA subjects [21].

Both SOS and ESS are the questionnaires that help decide whether a patient has a sleep problem. ESS measures daytime sleepiness and is often used clinically to screen for manifestations of behavioral morbidity associated with OSA [22]. SOS, in comparison, is another recently described questionnaire for evaluating patients with snoring problems. Although SOS is a subjective instrument, it is valid, reliable, and sensitive to clinical changes [23]. These questionnaires are effective in determining whether a patient has OSA problems; however, they are not helpful in determining the severity of sleep apnea. All patients were administered with the Chinese versions of SOS and ESS as the laboratory test routine. All surveys were validated and considered statistically equivalent to their original English versions [24, 25]. For ESS range 0–24, higher is worse. For SOS 0–100, higher is better.

Gender as a factor has only been recognized recently. Several studies have tried to provide an explanation for the male predominance in OSA, including differences in anatomical size, greater collapsibility of the upper airway, greater increase in upper airway resistance in men, and hormonal changes in women [17, 26]. However, gender is not helpful in determining the severity of sleep apnea.

BMI is a statistical measurement that compares weight and height. It is considered a useful index to estimate the body's level of obesity. Obesity is often seen in OSA patients, yet, in experimental results, BMI is not an important feature. The reason is that BMI is routinely used in PSG lab; therefore we checked this feature. However, our data show there is poor correlation between BMI and OSA severity; as a result, BMI is not included in the MMTS model to predict OSA.

PLM represents the frequency of paroxysmal leg movements per hour during night sleep and indicates the severity of sleep disturbance caused by this particular disease. A higher PLM contributes to worse situation.

### 5.2. Methods

To illustrate the effectiveness of MMTS for OSA prediction, comparisons were made between MMTS and other methods, including LR, BPN, LVQ, SVM, C4.5 decision tree, and RS. The observation made on the MMTS is significantly better than that of other classifications of algorithms. On the other hand, from the viewpoint of implementation, MMTS does not require any parameters to optimize its execution, whereas other techniques such as BPN and SVM consume much time in fine-tuning the parameters. The performance of these parameter-attached classification or feature selection techniques is always sensitive to the parameter determination. Effectively determining the best combination of parameter settings to optimize algorithm output remains a pending issue.

## 6. Conclusions

In recent years, OSA has become an important public health concern. A complete and thorough sleep checkup has to be conducted in a sleep laboratory or medical center, and the patient has to undergo the PSG test in a particular bed for the entire night. Various sensory devices are used on the patient to monitor overnight physical conditions, allowing the complete sleeping structure to be observed and any unusual sleeping condition to be detected. Doctors use the information obtained as the basis for diagnosis. The numerous steps in the PSG test to diagnose OSA are thus costly and time consuming. In this study which applies MMTS, the patient simply needs to wear the monitoring systems (e.g., oximeter) around the wrist like a watch and conduct an at-home overnight test. The monitoring systems are connected to a sensor wire clip placed on a fingertip. The obtained data are used in MMTS to anticipate the OSA diagnosis. Therefore, because it is extremely simple and convenient, this method can be useful for doctors in predicting an OSA diagnosis in advance before running the PSG test, allowing for a more effective use of medical resources.

## Figures and Tables

**Table 1:** The OSA attributes.

No.	Item.	Description
A	Gender	Gender (1, 2)^1^
B	Age	Years (0–100)
C	BW	Weight (Body weight, in kg)
D	BH	Height (body height, in cm)
E	BMI	Body Mass Index (body mass index, in kg/m^2^)
F	SBP	Systolic blood pressure (mm Hg)
G	DBP	Diastolic blood pressure (mm Hg)
H	ESS	Daytime sleepiness survey scale (0–24, 24=worst daytime sleepiness situation)
I	SOS	Snoring survey score (0–100, 0=Worst snoring score)
J	DI3	Frequency of desaturation (saturation index <3% in an hour)
K	DI4	Frequency of desaturation (saturation index <4% in an hour)
L	PLM	Frequency of paroxysmal leg movement in an hour

^1^1: Male, 2: Female.

**Table 2:** Demographic data, *N* = 86.

	Mean	Median	SD^1^	Range
Gender	1.23	1	0.42	1-2
Age	48.3	49	11.87	11–78
height	165.98	167	7.34	151–184
weight	69.04	68	11.31	49–116
BMI	24.98	24.78	2.13	18.34–34.26
SBP	124.64	122.5	17.62	83–178
DBP	81.23	81	10.46	53–108
ESS	10.07	11	6.38	0–24
SOS	50.23	46	21.20	18–95
DI3	92.76	37.5	121.66	0–550
DI4	92.47	36	121.70	0–550
PLM	2.72	0	8.68	0–47.1

^1^SD: Standard Deviation.

**Table 3:** The OSA data.

	Nondisease (normal)	Disease (mild)	Disease (moderate)	Disease (severe)	Total
Group I (training data)	16	23	10	8	57
Group II (testing data)	8	6	9	6	29

**Table 4:** A comparison.

Method	Selected attributes	Pattern	Accuracy (%)	
MMTS	Age, weight, SBP, DBP, DI3 DI4	normal	87.5%	Average
		mild	66.67%	84.38%
		moderate	100%	
		severe	83.33%	

Logistic Regression	Gender, age, height, weight, BMI, SBP, DBP, ESS, SOS, DI3, DI4, PLM	normal	50.00%	55.33%
		mild	50.00%	
		moderate	33.33%	
		severe	100.00%	

BPN	Gender, age, height, weight, BMI, SBP, DBP, ESS, SOS, DI3, DI4, PLM	normal	25.00%	34.04%
		mild	33.33%	
		moderate	11.11%	
		severe	66.70%	

LVQ	Gender, age, height, weight, BMI, SBP, DBP, ESS, SOS, DI3, DI4, PLM	normal	50.00%	47.22%
		mild	16.67%	
		moderate	22.22%	
		severe	100.00%	

SVM	Age, height, weight, BMI, SBP, DBP, ESS, SOS, DI3, DI4, PLM	normal	37.50%	53.82%
		mild	66.67%	
		moderate	11.11%	
		severe	100.00%	

C4.5	Age, weight, BMI, SBP, SOS, DI4	normal	37.50%	63.54%
		mild	50.00%	
		moderate	66.67%	
		severe	100.00%	

RS	Gender, Age, weight, SBP, ESS, SOS	normal	25.00%	13.20%
		mild	16.67%	
		moderate	11.11%	
		severe	0.00%	

## References

[1] National Sleep Foundation http://www.sleepfoundation.org/.

[2] Kirby SD, Danter W, George CFP, Francovic T, Ruby RRF, Ferguson KA (1999). Neural network prediction of obstructive sleep apnea from clinical criteria. *Chest*.

[3] Sharma SK, Kurian S, Malik V (2004). A stepped approach for prediction of obstructive sleep apnea in overtly asymptomatic obese subjects: a hospital based study. *Sleep Medicine*.

[4] Gurubhagavatula I, Maislin G, Pack AI (2001). An algorithm to stratify sleep apnea risk in a sleep disorders clinic population. *American Journal of Respiratory and Critical Care Medicine*.

[5] Jacob SV, Morielli A, Mograss MA, Ducharme FM, Schloss MD, Brouillette RT (1995). Home testing for pediatric obstructive sleep apnea syndrome secondary to adenotonsillar hypertrophy. *Pediatric pulmonology*.

[6] Yamashiro Y, Kryger MH (1995). Nocturnal oximetry: is it a screening tool for sleep disorders?. *Sleep*.

[7] Taguchi G, Chowdhury S, Wu Y (2001). *The Mahalanobis-Taguchi System*.

[8] Taguchi G, Jugulum R (2002). *The Mahalanobis-Taguchi Strategy*.

[9] Srinivasaraghavan J, Allada V (2006). Application of mahalanobis distance as a lean assessment metric. *International Journal of Advanced Manufacturing Technology*.

[10] Riho T, Suzuki A, Oro J, Ohmi K, Tanaka H (2005). The yield enhancement methodology for invisible defects using the MTS+ method. *IEEE Transactions on Semiconductor Manufacturing*.

[11] Das P, Datta S (2007). Exploring the effects of chemical composition in hot rolled steel product using Mahalanobis distance scale under Mahalanobis-Taguchi system. *Computational Materials Science*.

[12] Taguchi G, Chowdhury S, Wu Y (2005). *Taguchi’s Quality Engineering Handbook*.

[13] Su CT, Hsiao YH (2007). An evaluation of the robustness of MTS for imbalanced data. *IEEE Transactions on Knowledge and Data Engineering*.

[14] Su CT, Hsiao YH (2009). Multiclass MTS for simultaneous feature selection and classification. *IEEE Transactions on Knowledge and Data Engineering*.

[15] Brown M, Gunn SR, Lewis HG (1999). Support vector machines for optimal classification and spectral unmixing. *Ecological Modelling*.

[16] Matsumoto Y, Watada J (2009). Knowledge acquisition from time series data through rough sets analysis. *International Journal of Innovative Computing, Information and Control*.

[17] Kapsimalis F, Kryger MH (2002). Gender and obstructive sleep apnea syndrome—part 2: mechanisms. *Sleep*.

[18] Hirshkowitz M, Kryger MH, Kryger MH, Roth T, Dement WC (2005). Monitoring techniques for evaluating suspected sleep-disordered breathing. *Principle and Practice of Sleep Medicine*.

[19] Dixon JB, Schachter LM, O’Brien PE (2003). Predicting sleep apnea and excessive day sleepiness in the severely obese: indicators for polysomnography. *Chest*.

[20] Sharma SK, Malik V, Vasudev C (2006). Prediction of obstructive sleep apnea in patients presenting to a tertiary care center. *Sleep and Breathing*.

[21] Schäfer H, Ewig S, Hasper E, Lüderitz B (1997). Predictive diagnostic value of clinical assessment and nonlaboratory monitoring system recordings in patients with symptoms suggestive of obstructive sleep apnea syndrome. *Respiration*.

[22] Rosenthal LD, Dolan DC (2008). The Epworth sleepiness scale in the identification of obstructive sleep apnea. *Journal of Nervous and Mental Disease*.

[23] Gliklich RE, Wang PC (2002). Validation of the snore outcomes survey for patients with sleep-disordered breathing. *Archives of Otolaryngology*.

[24] Chen NH, Li HY, Gliklich RE, Chu CC, Liang SC, Wang PC (2002). Validation assessment of the Chinese version of the Snore Outcomes Survey. *Quality of Life Research*.

[25] Chen NH, Johns MW, Li HY (2002). Validation of a Chinese version of the Epworth sleepiness scale. *Quality of Life Research*.

[26] Shepertycky MR, Banno K, Kryger MH (2005). Differences between men and women in the clinical presentation of patients diagnosed with obstructive sleep apnea syndrome. *Sleep*.

